# Stress-Based FEM in the Problem of Bending of Euler–Bernoulli and Timoshenko Beams Resting on Elastic Foundation

**DOI:** 10.3390/ma14020460

**Published:** 2021-01-19

**Authors:** Zdzisław Więckowski, Paulina Świątkiewicz

**Affiliations:** Department of Mechanics of Materials, Łódź University of Technology, 90-924 Łódź, Poland; Paulina.Swiatkiewicz@p.lodz.pl

**Keywords:** finite element method, stress-based approach, Euler–Bernoulli beam, Timoshenko beam, shear locking

## Abstract

The stress-based finite element method is proposed to solve the static bending problem for the Euler–Bernoulli and Timoshenko models of an elastic beam. Two types of elements—with five and six degrees of freedom—are proposed. The elaborated elements reproduce the exact solution in the case of the piece-wise constant distributed loading. The proposed elements do not exhibit the shear locking phenomenon for the Timoshenko model. The influence of an elastic foundation of the Winkler type is also taken into consideration. The foundation response is approximated by the piece-wise constant and piece-wise linear functions in the cases of the five-degrees-of-freedom and six-degrees-of-freedom elements, respectively. An a posteriori estimation of the approximate solution error is found using the hypercircle method with the addition of the standard displacement-based finite element solution.

## 1. Introduction

Beams are commonly used in engineering practice; thus, they often are a matter of interest of advanced research and numerous papers. The Euler–Bernoulli beam theory is the most basic theory applied to slender beams that is based on assumption that straight lines normal to the mid-plane before deformation remain straight and normal to it after deformation. The effect of the transverse shear deformation is not taken into account. The theory that includes the transverse shear strain is the Timoshenko beam theory, which can be used for the analysis of thick or moderately thick beams. It requires introduction of the shear correction coefficient, which depends on the geometry of the cross-section of the beam (e.g., [1]). These two theories and other higher-order theories are introduced, e.g., in [2].

The Timoshenko beam theory is prone to the shear locking phenomenon in the course of numerical calculations by the finite element method (FEM). There exist methods that alleviate this problem. One of them is the linked interpolation technique introduced by Fraeijs de Veubeke [3] and described in detail in the book by Zienkiewicz and Taylor [4]. Among other methods eliminating the shear locking phenomenon, we may enumerate the reduced integration technique, the use of the higher-order theories of beam bending, the assumed shear strain technique, or the appropriate polynomial interpolation for deflection and rotation of the beam. The reduced integration [5,6] was used, e.g., by Yokoyama [7] in problem of the Timoshenko beam vibration analysis. The higher-order beam bending theory was applied, e.g., by Reddy et al. [8]. Mukherjee et al. [9] utilized the assumed strain approach. The polynomial interpolation for the displacement that is one degree higher than that used for the rotation may be found, e.g., in [10,11]. Some recent studies that circumvent shear locking also include one by Kanok-Nukulchai et al. [12] that concerns element-free Galerkin method in conjunction with moving weighted least-squares approximation (also used by Beissel and Belytschko [13], and Belytschko, Lu and Gu [14]), the works based on meshless analysis (e.g., [15]), or the paper by Feng, Cui and Li [16] that concerns a nodal integration technique for static and dynamic problems of Timoshenko beams. Further research on this topic may be found in books [4,11,17].

Recently, the Timoshenko beam was analyzed by numerous authors using the isogeometric discretization approach. Papers by Beirão de Veiga et al. [18] and Auricchio et al. [19] present how to avoid shear locking in the case of the Timoshenko beam problem using the isogeometric collocation method. Kiendl et al. [20] introduced an element with the rotation as the primal variable and used isogeometric collocation as well. Balobanov and Niiranen [21] described Timoshenko beam bending on the basis of the strain gradient elasticity theory.

The strain gradient elasticity theory was also the formulation basis for static, dynamic, and buckling analyses of the Timoshenko microbeams studied by Zhang et al. [22]. Wong and Sugianto [23] formulated the discrete shear gap technique in the case of linear, quadratic, and cubic Timoshenko beam elements.

Analysis of beams resting on elastic foundation is another field of interest of researchers. One of the basic models for elastic response of the foundation is the Winkler foundation, which may be found, e.g., in the work by Heteneyi [24].

The aim of this paper is to present the stress-based approach to Euler–Bernoulli and Timoshenko beams that are competitive to the well-known displacement-based models. The proposed method is efficient and does not suffer from shear locking. The introduced models include the elastic foundation of Winkler type. The obtained results are convergent and enable us to obtain the upper bound to the strain energy when only external forces are prescribed. It is also easy to estimate the error of the approximate solution when the displacement-based and the stress-based models are concurrently applied. The stress-based approach to the beam bending problems is not popular in the literature and engineering practice. An exception is the paper by Kuo et al. [25] describing an attempt of construction of the stress-based finite element model in the case of the Euler–Bernoulli beam. It seems, however, that in this paper the statically admissible stresses were built only in the case of absence of a distributed load.

The paper is divided into seven sections. The stress-based formulations of the Euler–Bernoulli and Timoshenko beams are developed in Section 2, Section 3 and Section 4. Section 5 deals with estimation of the error of approximate solution based on kinematically and statically admissible solutions. Section 6 gives some numerical results. Concluding remarks are gathered in Section 7.

## 2. Stress-Based Formulation of the Euler–Bernoulli Beam on Elastic Foundation

### 2.1. Governing Equations

In the case of the Euler–Bernoulli beam resting on Winkler elastic foundation, the governing differential equations under static loading conditions for $x\in \left(0,l\right)$ can be expressed as follows (e.g., [8,11]):

(1) $$\begin{array}{c} \frac{dM(x)}{dx}=Q\left(x\right),\;\frac{dQ(x)}{dx}=-q\left(x\right)+q_{r}\left(x\right), \end{array}$$

(2) $$\begin{array}{c} -\frac{d^{2}w\left(x\right)}{dx^{2}}=\kappa \left(x\right), \end{array}$$

(3) $$\begin{array}{c} M(x)=EJ\;\kappa (x), \end{array}$$

where M(x) and Q(x) are the bending moment and the shear force functions respectively, q(x) denotes the intensity of the transverse distributed load, and $q_{r}\left(x\right)$ is the foundation response. Function w(x) represents the transverse displacement of the beam, κ(x) denotes the curvature of the beam, *E* is the Young modulus, and *J* the inertia moment of the cross-section area. In the Winkler elastic foundation model, $q_{r}\left(x\right)$ is assumed to be linearly dependent on the beam deflection

(4) $$\begin{array}{c} q_{r}\left(x\right)=k\;w\left(x\right), \end{array}$$

where *k* is a positive material constant. The governing differential Equations (1)–(3) are complemented with boundary conditions. These may be of static nature

(5) $$\begin{array}{c} M=\bar{M}\;\mathrm{on}\;\Gamma_{M},\;Q=\bar{Q}\;\mathrm{on}\;\Gamma_{Q}, \end{array}$$

or kinematic nature

(6) $$\begin{array}{c} w=\bar{w}\;\mathrm{on}\;\Gamma_{w},\;\frac{dw}{dx}=\bar{\varphi }\;\mathrm{on}\;\Gamma_{\varphi }, \end{array}$$

where the overbared terms mean given values of particular quantities. $\Gamma_{M}$ and $\Gamma_{Q}$ denote the sets of the beam end-points, where moments and shear forces are given, respectively, and $\Gamma_{w}$ and $\Gamma_{\varphi }$ are the sets of end-points where deflections and their derivatives are specified, respectively. Each of sets $\Gamma_{M}$, $\Gamma_{Q}$, $\Gamma_{w}$, and $\Gamma_{\varphi }$ can be an empty set. It is noted that

$$\begin{array}{c} \Gamma_{M}\cap \Gamma_{\varphi }=\emptyset ,\;\Gamma_{Q}\cap \Gamma_{w}=\emptyset . \end{array}$$

### 2.2. The Complementary Work Principle

Let us define the following generalized stress, σ, and strain, ε, vectors:

(7) $$\begin{array}{c} \sigma =\left[\begin{array}{c} M\\ q_{r} \end{array}\right],\;\varepsilon =\left[\begin{array}{c} \kappa \\ w \end{array}\right]=\left[\begin{array}{c} -\frac{d^{2}w}{dx^{2}}\\ w \end{array}\right]. \end{array}$$

Let the following set of statically admissible functions of the generalized stress be defined:

(8) $$\begin{array}{c} Y_{\bar{M},\bar{Q},q}=\left\{\sigma \in {\left[L^{2}\left[0,l\right]\right]}^{2}:\;\frac{dQ}{dx}=-q+q_{r},\;\frac{dM}{dx}=Q,\;M=\bar{M}\;\mathrm{on}\;\Gamma_{M},\;Q=\bar{Q}\;\mathrm{on}\;\Gamma_{Q}\right\}, \end{array}$$

and let the following space of statically admissible generalized stress be defined:

(9) $$\begin{array}{c} Y_{0,0,0}=\left\{\sigma \in {\left[L^{2}\left[0,l\right]\right]}^{2}:\;\frac{dQ}{dx}=q_{r},\;\frac{dM}{dx}=Q,\;M=0\;\mathrm{on}\;\Gamma_{M},\;Q=0\;\mathrm{on}\;\Gamma_{Q}\right\}, \end{array}$$

where $L^{2}\left[0,l\right]$ means the space of square integrable functions in the interval [0,l]. The weak formulation of the problem is obtained by multiplication of geometric relation (2) by a variation in bending moment and the subsequent integration of the result over the length of the beam

(10) $$\begin{array}{c} \int_{0}^{l}\left(\kappa +\frac{d^{2}w}{dx^{2}}\right)\delta M\;dx=0\;\forall \delta M\in L^{2}\left[0,l\right]. \end{array}$$

After use of integration by parts and inserting Equations (3) and (4), the above equation can be rewritten as

(11) $$\begin{array}{c} \int_{0}^{l}M\;\frac{1}{EJ}\;\delta M\;dx+\int_{0}^{l}q_{r}\;\frac{1}{k}\;\delta q_{r}\;dx+{\left[\frac{dw}{dx}\;\delta M\right]}_{0}^{l}-{\left[w\;\frac{d\;\delta M}{dx}\right]}_{0}^{l}=0\;\forall \;\delta \sigma \in {\left[L^{2}\left[0,l\right]\right]}^{2}. \end{array}$$

The principle of complementary work can be formulated as follows. Find $M\in Y_{\bar{M},\bar{Q},q}$ such that the following equation is satisfied:

(12) $$\begin{array}{c} \int_{0}^{l}M\;\frac{1}{EJ}\;\delta M\;dx+\int_{0}^{l}q_{r}\;\frac{1}{k}\;\delta q_{r}\;dx+\bar{\varphi }\;\delta M{|}_{\Gamma_{\varphi }}-\bar{w}\;\delta Q{|}_{\Gamma_{w}}=0\;\forall \delta \sigma \in Y_{0,0,0}. \end{array}$$

The aforementioned formulation is equivalent to the problem of minimizing the complementary energy functional on set $Y_{\bar{M},\bar{Q},q}$:

(13) $$\begin{array}{c} \Sigma \left(\sigma \right)=\frac{1}{2}\int_{0}^{l}\frac{1}{EJ}\;M^{2}\;dx+\frac{1}{2}\int_{0}^{l}\frac{1}{k}\;q_{r}^{2}\;dx+\bar{\varphi }\;M{|}_{\Gamma_{\varphi }}-\bar{w}\;Q{|}_{\Gamma_{w}}. \end{array}$$

### 2.3. Matrix Form of the Equilibrium-Based Fe Method

A numerical model of the Euler–Bernoulli beam is established assuming that the equilibrium conditions are satisfied exactly. In order to satisfy the equilibrium equations along the beam element, we assume that the bending moment is approximated with use of the matrix of linear shape functions $N_{M}\equiv \left[\begin{array}{cc} N_{1} & N_{2} \end{array}\right]$, the vector of its nodal values $a_{M}\equiv {\left[\begin{array}{cc} M_{1} & M_{2} \end{array}\right]}^{T}$, and two additional terms $M_{q}$ and $M_{r}$,

(14) $$\begin{array}{c} M=N_{M}\;a_{M}+M_{r}-M_{q}. \end{array}$$

$M_{q}\equiv \int \int q\left(x\right)\;dx\;dx$ is a function which imposes equilibrium when the transverse distributed load is applied to the beam. In the case of constant load, it can be expressed in the form of the polynomial of the second degree. Term $M_{r}$ describes the influence of the elastic foundation on the bending moment function. The foundation response is approximated element-wise with use of a constant function. Consequently, to satisfy equilibrium (Equation (1)) along the element, term $M_{r}$ requires insertion of quadratic shape function ${\tilde{N}}_{e}=\frac{1}{2}\;x\;\left(l_{e}-x\right)$ and $q_{re}$ as a degree of freedom defined at a node located inside the *e*th element. The specific expressions for the approximation of the generalized stress vector, σ (the bending moment and the foundation response), can be written as follows:

(15) $$\begin{array}{c} \sigma =\left[\begin{array}{c} M\\ q_{r} \end{array}\right]=\left[\begin{array}{cccccc} N_{1} & {\tilde{N}}_{1} & N_{2} & {\tilde{N}}_{2} & N_{3} & \ldots \\ 0 & 1 & 0 & 1 & 0 & \ldots \end{array}\right]\;\left[\begin{array}{c} M_{1}\\ q_{r1}\\ M_{2}\\ q_{r2}\\ M_{3}\\ \vdots \\ q_{rn_{e}}\\ M_{n_{n}} \end{array}\right]-\left[\begin{array}{c} M_{q}\\ 0 \end{array}\right]\equiv N\;a-M_{q}, \end{array}$$

where indices $n_{e}$ and $n_{n}$ denote the numbers of elements and nodes, respectively. Substitution of Equation (15) into Equation (12) leads to the following matrix form of the complementary work principle:

(16) $$\begin{array}{c} \delta a^{T}\;\left(\int_{0}^{l}N^{T}\;C\;\left(N\;a-M_{q}\right)\;dx+f\right)=0\;\;\forall \;\delta a\;\mathrm{such}\;\mathrm{that}\;N\;a\in Y_{0,0,0}, \end{array}$$

where

(17) $$\begin{array}{c} C=\left[\begin{array}{cc} \frac{1}{EJ} & 0\\ 0 & \frac{1}{k} \end{array}\right]. \end{array}$$

and vector f contains zeros except for the terms related to the degrees of freedom defined at the ends of the beam in the case of $\bar{w}\neq 0$ and $\bar{\varphi }\neq 0$. Satisfaction of Equation (16) implies the following set of algebraic equations:

(18) $$\begin{array}{c} Ka=F, \end{array}$$

where K denotes the global compliance matrix and F stands for the global right-hand-side terms vector

(19) $$\begin{array}{c} K=\int_{0}^{l}N^{T}\;C\;N\;dx,\;\;F=\int_{0}^{l}N^{T}\;C\;M_{q}\;dx+f. \end{array}$$

### 2.4. Interelement Equilibrium

**Figure 1:**
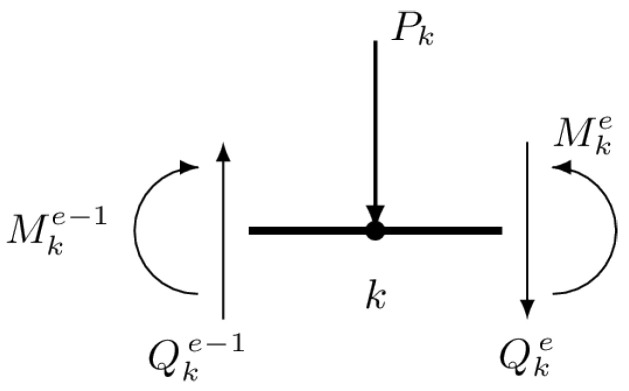
Equilibrium of bending moments and lateral forces at the interelement node.

Appropriate selection of interpolation functions guarantees equilibrium inside each of the elements. However, choosing linear shape functions for the bending moment does not guarantee continuity of the shear force between the elements. Thus, in order to fully satisfy the equilibrium equations it is necessary to impose additional conditions for the shear force at interelement nodes. The equilibrium of the interelement node of the beam (Figure 1) is ensured provided that

(20) $$\begin{array}{c} P_{k}=-Q_{k}^{\;e}+Q_{k}^{\;e-1}, \end{array}$$

where $P_{k}$ denotes the value of the concentrated load applied to the *k*th node. Expressions for forces $Q_{k}^{\;e-1}$ and $Q_{k}^{\;e}$ are derived by differentiation of the bending moment function

(21) $$\begin{array}{c} Q=\frac{dM}{dx}=\frac{dN}{dx}a. \end{array}$$

To satisfy the system (18) with constraints (20), the following enhanced Lagrange function is considered:

(22) $$\begin{array}{c} \bar{\Sigma }\left(a,\lambda \right)=\frac{1}{2}\;a^{T}\;K\;a-a^{T}\;F+\sum_{k=1}^{n_{i}}\lambda_{k}\;\left(-Q_{k}^{\;e}+Q_{k}^{\;e-1}-P_{k}\right), \end{array}$$

where $n_{i}$ stands for the number of internal nodes and $\lambda_{k}$ are Lagrange multipliers. Applying the multipliers is effective in this particular case not only because the required constraints are enforced but also due to their physical interpretation as displacement of the nodes. It should be pointed out that the stress-based quantities (approximate functions of bending moment and foundation response) as well as displacement-based ones (nodal deflections) are known as a result.

### 2.5. Applied Element

**Figure 2:**
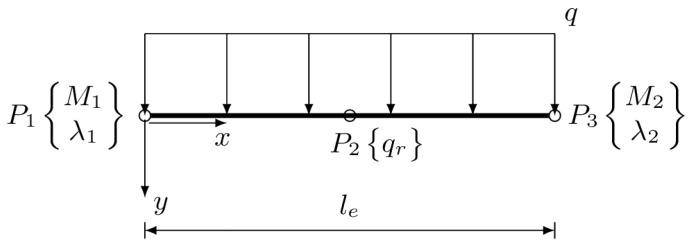
Three-node, five-degrees-of-freedom stress-based beam element.

Element of class $C^{0}$ with linear shape functions is applied in the present paper. It can be loaded with uniform distributed load *q*. It has three nodes and five degrees of freedom, as shown in Figure 2.

The vector of element degrees of freedom is therefore $a_{e}={\left[\begin{array}{ccccc} M_{1} & \lambda_{1} & q_{r} & M_{2} & \lambda_{2} \end{array}\right]}^{T}$.

The element is equipped with the following shape functions:

(23) $$\begin{array}{c} N_{e}=\left[\begin{array}{ccccc} N_{1} & 0 & \tilde{N} & N_{2} & 0\\ 0 & 0 & 1 & 0 & 0 \end{array}\right] \end{array}$$

where $N_{1}=1-\frac{x}{l_{e}}$ and $N_{2}=\frac{x}{l_{e}}$ are linear shape functions corresponding to $M_{1}$ and $M_{2}$, respectively, whereas $\tilde{N}=\frac{1}{2}\;x\left(l_{e}-x\right)$ is related to the influence of the foundation response, $q_{r}$, on the bending moment function. The generalized stress vector that contains bending moment and foundation response is approximated with use of matrix of shape functions $N_{e}$, vector of degrees of freedom $a_{e}$, and a vector containing term $M_{q}$

(24) $$\begin{array}{c} \sigma =\left[\begin{array}{c} M\\ q_{r} \end{array}\right]=N_{e}\;a_{e}-\left[\begin{array}{c} M_{q}\\ 0 \end{array}\right], \end{array}$$

where

(25) $$\begin{array}{c} M_{q}=\frac{q\;x\;(l_{e}-x)}{2} \end{array}$$

is the term corresponding to the distributed load. In the case of the Euler–Bernoulli beam, the element matrix derived from (19) can be expressed explicitly:

(26) $$\begin{array}{c} k_{e}^{\mathrm{EB}}=\left[\begin{array}{ccccccccc} \int_{0}^{l_{e}}\frac{1}{EJ}\;N_{1}\;N_{1}\;dx & \; & \frac{1}{l_{e}} & \; & \int_{0}^{l_{e}}\frac{1}{EJ}\;N_{1}\;\tilde{N}\;dx & \; & \int_{0}^{l_{e}}\frac{1}{EJ}\;N_{1}\;N_{2}\;dx & \; & -\frac{1}{l_{e}}\\ \frac{1}{l_{e}} & \; & 0 & \; & \frac{1}{2}\;l_{e} & \; & -\frac{1}{l_{e}} & \; & 0\\ \int_{0}^{l_{e}}\frac{1}{EJ}\;N_{1}\;\tilde{N}\;dx & \; & \frac{1}{2}\;l_{e} & \; & \int_{0}^{l_{e}}\frac{1}{EJ}\;\tilde{N}\;\tilde{N}\;dx+\int_{0}^{l_{e}}\frac{1}{k}\;\frac{d^{2}\tilde{N}}{dx^{2}}\;\frac{d^{2}\tilde{N}}{dx^{2}}\;dx & \; & \int_{0}^{l_{e}}\frac{1}{EJ}\;\tilde{N}\;N_{2}\;dx & \; & \frac{1}{2}\;l_{e}\\ \int_{0}^{l_{e}}\frac{1}{EJ}\;N_{1}\;N_{2}\;dx & \; & -\frac{1}{l_{e}} & \; & \int_{0}^{l_{e}}\frac{1}{EJ}\;\tilde{N}\;N_{2}\;dx & \; & \int_{0}^{l_{e}}\frac{1}{EJ}\;N_{2}\;N_{2}\;dx & \; & \frac{1}{l_{e}}\\ -\frac{1}{l_{e}} & \; & 0 & \; & \frac{1}{2}\;l_{e} & \; & \frac{1}{l_{e}} & \; & 0 \end{array}\right] \end{array}$$

and the element right-hand-side terms vector, where *q* is taken into consideration, is

(27) $$\begin{array}{c} f_{e}^{\mathrm{EB}}=\left[\begin{array}{c} \int_{0}^{l_{e}}\frac{1}{EJ}\;N_{1}\;M_{q}\;dx\\ q\;\frac{l_{e}}{2}\\ \int_{0}^{l_{e}}\frac{1}{EJ}\;\tilde{N}\;M_{q}\;dx\\ \int_{0}^{l_{e}}\frac{1}{EJ}\;N_{2}\;M_{q}\;dx\\ q\;\frac{l_{e}}{2} \end{array}\right]. \end{array}$$

The second and fifth rows of matrix (26), having zeros on the main matrix diagonal, are related to Lagrange multipliers $\lambda_{1}$ and $\lambda_{2}$.

## 3. Stress-Based Formulation for the Timoshenko Beam on Elastic Foundation

### 3.1. Governing Equations and Weak Formulation

In the case of the Timoshenko beam model, the shear deformation caused by the shear force is taken into consideration. The measure of this deformation, angle γ, depends on two kinematic quantities: the deflection, *w*, and the cross-section rotation, φ, that are independent of each other. The set of equations describing the static bending problem of the Timoshenko beam can be written for x∈(0,l) as follows (e.g., [8,9,11,15,16,20]):

(28) $$\begin{array}{c} \frac{dM(x)}{dx}=Q\left(x\right),\;\frac{dQ(x)}{dx}=-q\left(x\right)+q_{r}\left(x\right), \end{array}$$

(29) $$\begin{array}{c} \gamma \left(x\right)=-\varphi \left(x\right)+\frac{dw(x)}{dx} \end{array}$$

(30) $$\begin{array}{c} M\left(x\right)=-EJ\;\frac{d\varphi (x)}{dx},\;Q\left(x\right)=K_{s}\gamma \left(x\right), \end{array}$$

where $K_{s}$ denotes the shear stiffness of the beam cross-section, which can be expressed as

(31) $$\begin{array}{c} K_{s}=\frac{G\;A}{\beta }, \end{array}$$

where *G* is the shear modulus, *A* is the cross-section area, and β denotes the coefficient dependent on the cross-section shape. Other symbols have the same meaning as in Section 2.1. The natural and essential boundary conditions that complement Equations (28)–(30) are given as

(32) $$\begin{array}{c} M=\bar{M}\;\mathrm{on}\;\Gamma_{M},\;Q=\bar{Q}\;\mathrm{on}\;\Gamma_{Q}, \end{array}$$

(33) $$\begin{array}{c} w=\bar{w}\;\mathrm{on}\;\Gamma_{w},\;\varphi =\bar{\varphi }\;\mathrm{on}\;\Gamma_{\varphi }, \end{array}$$

where the notation from Section 2.1 is utilized.

### 3.2. The Complementary Work Principle

Let us now consider the vectors of generalized stress and strain as follows:

(34) $$\begin{array}{c} \sigma =\left[\begin{array}{c} M\\ Q\\ q_{r} \end{array}\right],\;\varepsilon =\left[\begin{array}{c} \kappa \\ \gamma \\ w \end{array}\right]=\left[\begin{array}{c} -\frac{d\varphi }{dx}\\ \frac{dw}{dx}-\varphi \\ w \end{array}\right]. \end{array}$$

The set of the statically admissible generalized stress is defined as follows:

(35) $$\begin{array}{c} Y_{\bar{M},\bar{Q},q}=\left\{\sigma \in {\left[L^{2}\left[0,l\right]\right]}^{3}:\;\frac{dQ}{dx}=-q+q_{r},\;\frac{dM}{dx}=Q,\;M=\bar{M}\;\mathrm{on}\;\Gamma_{M},\;Q=\bar{Q}\;\mathrm{on}\;\Gamma_{Q}\right\}, \end{array}$$

and let the following space of statically admissible stress-related quantities be defined:

(36) $$\begin{array}{c} Y_{0,0,0}=\left\{\sigma \in {\left[L^{2}\left[0,l\right]\right]}^{3}:\;\frac{dQ}{dx}=q_{r},\;\frac{dM}{dx}=Q,\;M=0\;\mathrm{on}\;\Gamma_{M},\;Q=0\;\mathrm{on}\;\Gamma_{Q}\right\}. \end{array}$$

The complementary work principle can be obtained by multiplication of geometric relation Equation (29) by a variation in shearing force belonging to the space $Y_{0,0,0}$ and the subsequent integration over the length of the beam

(37) $$\begin{array}{c} \int_{0}^{l}\left(\gamma \left(x\right)+\varphi \left(x\right)-\frac{dw(x)}{dx}\right)\delta Q\;dx=0\;\forall \delta Q\in L^{2}\left(0,l\right). \end{array}$$

Then, the integration by parts and employment of Equations (4) and (30) is used. Therefore, Equation (37) can be rewritten as

(38) $$\begin{array}{c} \int_{0}^{l}M\;\frac{1}{EJ}\;\delta M\;dx+\int_{0}^{l}Q\;\frac{1}{K_{s}}\;\delta Q\;dx+\int_{0}^{l}q_{r}\;\frac{1}{k}\;\delta q_{r}\;dx+\bar{\varphi }\;\delta M{|}_{\Gamma_{\varphi }}-\bar{w}\;\delta Q{|}_{\Gamma_{w}}=0\;\forall \;\delta \sigma \in Y_{0,0,0}. \end{array}$$

The principle of complementary work can be formulated as follows. Find $\sigma \in Y_{\bar{M},\bar{Q},q}$ such that the following equation is satisfied:

(39) $$\begin{array}{c} \int_{0}^{l}M\;\frac{1}{EJ}\;\delta M\;dx+\int_{0}^{l}Q\;\frac{1}{K_{s}}\;\delta Q\;dx+\int_{0}^{l}q_{r}\;\frac{1}{k}\;\delta q_{r}\;dx=-\bar{\varphi }\;\delta M{|}_{\Gamma_{\varphi }}+\bar{w}\;\delta Q{|}_{\Gamma_{w}}\;\forall \;\delta \sigma \in Y_{0,0,0}. \end{array}$$

The above formulation is equivalent to the problem of minimizing the complementary energy functional on set $Y_{\bar{M},\bar{Q},q}$:

(40) $$\begin{array}{c} \Sigma \left(\sigma \right)=\frac{1}{2}\left(\int_{0}^{l}\frac{1}{EJ}\;M^{2}\;dx+\int_{0}^{l}\frac{1}{K_{s}}\;Q^{2}\;dx+\int_{0}^{l}\frac{1}{k}\;q_{r}^{2}\;dx\right)+\bar{\varphi }\;M{|}_{\Gamma_{\varphi }}-\bar{w}\;Q{|}_{\Gamma_{w}}. \end{array}$$

### 3.3. Finite Element Formulation of the Stress-Based Approach

In the Timoshenko beam problem, the statically admissible functions of the bending moment and the foundation reaction are constructed in the same way as for the Euler–Bernoulli model described in Section 2.3. The additional component of the stress vector, the shearing force, is determined by the equilibrium Equation (28)${}_{1}$. Thus, the matrix form of the approximation of the stress vector components becomes

(41) $$\begin{array}{c} \sigma =\left[\begin{array}{c} M\\ Q\\ q_{r} \end{array}\right]=\left[\begin{array}{cccccc} N_{1} & {\tilde{N}}_{1} & N_{2} & {\tilde{N}}_{2} & N_{3} & \ldots \\ \frac{dN_{1}}{dx} & \frac{d{\tilde{N}}_{1}}{dx} & \frac{dN_{2}}{dx} & \frac{d{\tilde{N}}_{2}}{dx} & \frac{dN_{3}}{dx} & \ldots \\ 0 & 1 & 0 & 1 & 0 & \ldots \end{array}\right]\;\left[\begin{array}{c} M_{1}\\ q_{r1}\\ M_{2}\\ q_{r2}\\ M_{3}\\ \vdots \end{array}\right]-\left[\begin{array}{c} M_{q}\\ \frac{dM_{q}}{dx}\\ 0 \end{array}\right]\equiv N\;a-M_{q}. \end{array}$$

Using the matrix notation and substitution of Equation (41) into Equation (39), the complementary work principle can be written as

(42) $$\begin{array}{c} \delta a^{T}\;\int_{0}^{l}N^{T}\;C\;\left(N\;a-M_{q}\right)\;dx=0\;\;\forall \;\delta a\;\mathrm{such}\;\mathrm{that}\;N\;a\in Y_{0,0,0}, \end{array}$$

where C is the compliance matrix,

(43) $$\begin{array}{c} C=\left[\begin{array}{ccc} \frac{1}{EJ} & 0 & 0\\ 0 & \frac{1}{K_{s}} & 0\\ 0 & 0 & \frac{1}{k} \end{array}\right]. \end{array}$$

Thus, the vector of degrees of freedom, a, satisfies the following set of algebraic equations:

(44) $$\begin{array}{c} Ka=F, \end{array}$$

where

(45) $$\begin{array}{c} K=\int_{0}^{l}N^{T}\;C\;N\;dx,\;\;F=\int_{0}^{l}N^{T}\;C\;M_{q}\;dx. \end{array}$$

It is seen that the term, $\int_{0}^{l}\;Q/K_{s}\;\delta Q\;dx$, is related with the same number and types of the degrees of freedom as in the case of the Euler–Bernoulli beam element. The differences between the two considered models appear only in vector σ and matrix C. The equilibrium conditions for shear forces at nodes are satisfied as described in Section 2.4. Therefore, the element matrix and the element right-hand-side vector derived from Equation (45) can be expressed explicitly:

(46) $$\begin{array}{c} k_{e}^{\mathrm{Tim}}=k_{e}^{\mathrm{EB}}+\left[\begin{array}{ccccc} \int_{0}^{l_{e}}\frac{1}{K_{s}}\frac{dN_{1}}{dx}\frac{dN_{1}}{dx}\;dx & 0 & \int_{0}^{l_{e}}\frac{1}{K_{s}}\frac{dN_{1}}{dx}\frac{d\tilde{N}}{dx}\;dx & \int_{0}^{l_{e}}\frac{1}{K_{s}}\frac{dN_{1}}{dx}\frac{dN_{2}}{dx}\;dx & 0\\ 0 & 0 & 0 & 0 & 0\\ \int_{0}^{l_{e}}\frac{1}{K_{s}}\frac{dN_{1}}{dx}\frac{d\tilde{N}}{dx}\;dx & 0 & \int_{0}^{l_{e}}\frac{1}{K_{s}}\frac{d\tilde{N}}{dx}\frac{d\tilde{N}}{dx}\;dx & \int_{0}^{l_{e}}\frac{1}{K_{s}}\;\frac{d\tilde{N}}{dx}\;\frac{dN_{2}}{dx}\;dx & 0\\ \int_{0}^{l_{e}}\frac{1}{K_{s}}\frac{dN_{1}}{dx}\frac{dN_{2}}{dx}\;dx & 0 & \int_{0}^{l_{e}}\frac{1}{K_{s}}\;\frac{d\tilde{N}}{dx}\;\frac{dN_{2}}{dx}\;dx & \int_{0}^{l_{e}}\frac{1}{K_{s}}\frac{dN_{2}}{dx}\frac{dN_{2}}{dx}\;dx & 0\\ 0 & 0 & 0 & 0 & 0 \end{array}\right], \end{array}$$

(47) $$\begin{array}{c} f_{e}^{\mathrm{Tim}}=f_{e}^{\mathrm{EB}}+\left[\begin{array}{c} \int_{0}^{l_{e}}\frac{1}{K_{s}}\;\frac{dN_{1}}{dx}\;\frac{dM_{q}}{dx}\;dx\\ 0\\ \int_{0}^{l_{e}}\frac{1}{K_{s}}\;\frac{d\tilde{N}}{dx}\;\frac{dM_{q}}{dx}\;dx\\ \int_{0}^{l_{e}}\frac{1}{K_{s}}\;\frac{dN_{2}}{dx}\;\frac{dM_{q}}{dx}\;dx\\ 0 \end{array}\right], \end{array}$$

where $k_{e}^{\mathrm{EB}}$ and $f_{e}^{\mathrm{EB}}$ are the corresponding compliance matrix (26) and right-hand-side vector (27) related with the Euler–Bernoulli beam element. Inserting the expressions for functions $N_{1}$, $N_{2}$, $\tilde{N}$, and $M_{q}$ in Equations (46) and (47) leads to the following forms of element matrices:

(48) $$\begin{array}{c} k_{e}^{\mathrm{Tim}}=\left[\begin{array}{ccccc} \frac{1}{3}\;\frac{l}{EJ} & \frac{1}{l} & \frac{1}{24}\;\frac{l^{3}}{\mathrm{EJ}} & \frac{1}{6}\;\frac{l}{EJ} & -\frac{1}{l}\\ \frac{1}{l} & 0 & -\frac{1}{2}\;l & -\frac{1}{l} & 0\\ \frac{1}{24}\;\frac{l^{3}}{EJ} & -\frac{1}{2}\;l & \frac{1}{120}\;\frac{l^{5}}{EJ}+\frac{l}{k} & \frac{1}{24}\;\frac{l^{3}}{EJ} & -\frac{1}{2}\;l\\ \frac{1}{6}\;\frac{l}{EJ} & -\frac{1}{l} & \frac{1}{24}\;\frac{l^{3}}{EJ} & \frac{1}{3}\;\frac{l}{EJ} & \frac{1}{l}\\ -\frac{1}{l} & 0 & -\frac{1}{2}\;l & \frac{1}{l} & 0 \end{array}\right]+\left[\begin{array}{ccccc} \frac{1}{K_{s}\;l} & 0 & 0 & -\frac{1}{K_{s}\;l} & 0\\ 0 & 0 & 0 & 0 & 0\\ 0 & 0 & \frac{1}{12}\;\frac{l^{3}}{K_{s}} & 0 & 0\\ -\frac{1}{K_{s}\;l} & 0 & 0 & \frac{1}{K_{s}\;l} & 0\\ 0 & 0 & 0 & 0 & 0 \end{array}\right], \end{array}$$

(49) $$\begin{array}{c} f_{e}^{\mathrm{Tim}}=\left[\begin{array}{c} -\frac{1}{24}\;\frac{ql^{3}}{EJ}\\ \frac{1}{2}\;ql\\ -\frac{1}{120}\;\frac{ql^{5}}{EJ}\\ -\frac{1}{24}\;\frac{ql^{3}}{EJ}\\ \frac{1}{2}\;ql \end{array}\right]+\left[\begin{array}{c} 0\\ 0\\ -\frac{1}{12}\;\frac{ql^{3}}{K_{s}}\\ 0\\ 0 \end{array}\right]. \end{array}$$

Although zero terms occur on the main diagonal of the compliance matrix, pivoting is not necessary if the nodal bending moment is eliminated before the Lagrange multiplier for each node in the process of solving the global system of equations. When the bending moment is fixed at a beam end node, starting node numbering from an internal node allows one to avoid occurring zeros during the elimination process of the unknowns.

The element described above is briefly named 2M1f (two nodes related with the bending moment and one node related with the foundation response) in the further part of the paper.

## 4. The Stress-Based Beam Element with Linear Interpolation of Foundation Response

**Figure 3:**
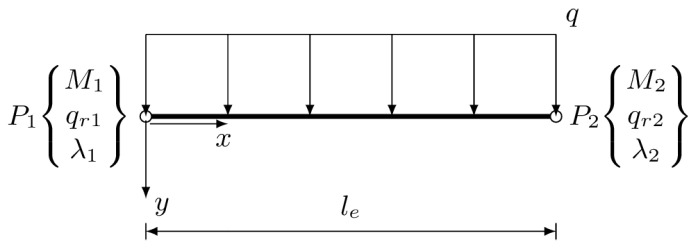
Stress-based beam element with two nodes and six degrees of freedom (2M2f).

A better performance of an equilibrium element with linear interpolation functions applied to approximation of the foundation response is expected. To implement this concept, the two node element with three degrees of freedom at each node is constructed. The degrees of freedom are the nodal values of the bending moment, the foundation response, and the Lagrange multiplier applied to satisfy the equilibrium of shear forces between elements. The proposed element is illustrated in Figure 3.

This element will be called 2M2f in the following part of the paper. Both the unknown functions of the bending moment and the foundation reaction are approximated with linear shape functions $N_{1}=1-\frac{x}{l_{e}}$ and $N_{2}=\frac{x}{l_{e}}$. These two functions—approximating the foundation response—are related through the equilibrium equations to the following bending moment functions:

(50) $$\begin{array}{c} {\tilde{N}}_{1}=-\frac{1}{6}\frac{x^{3}}{l_{e}}+\frac{1}{2}x^{2}-\frac{1}{3}l_{e}x,\;{\tilde{N}}_{2}=\frac{1}{6}\frac{x^{3}}{l_{e}}-\frac{1}{6}l_{e}x. \end{array}$$

These functions satisfy the homogeneous boundary conditions at the ends of the element.

It follows from the derivations shown in Section 2 and Section 3 that the element matrices can be represented by the part related to the Euler–Bernoulli beam model completed with the part containing terms related to the cross-section shear deformation existing in the case of the Timoshenko beam model. Therefore, further derivations will be conducted for the more general case of the Timoshenko beam element. The matrices for the Euler–Bernoulli beam element can be obtained by considering that $K_{s}\to \infty$.

The components of the generalised stress vector for a single element can be represented in a similar way as shown in Equation (41)

(51) $$\begin{array}{c} \sigma =\left[\begin{array}{c} M\\ Q\\ q_{r} \end{array}\right]=\left[\begin{array}{cccccc} N_{1} & {\tilde{N}}_{1} & 0 & N_{2} & {\tilde{N}}_{2} & 0\\ \frac{dN_{1}}{dx} & \frac{d{\tilde{N}}_{1}}{dx} & 0 & \frac{dN_{2}}{dx} & \frac{d{\tilde{N}}_{2}}{dx} & 0\\ 0 & N_{1} & 0 & 0 & N_{2} & 0 \end{array}\right]\;\left[\begin{array}{c} M_{1}\\ q_{r1}\\ \lambda_{1}\\ M_{2}\\ q_{r2}\\ \lambda_{2} \end{array}\right]-\left[\begin{array}{c} M_{q}\\ \frac{dM_{q}}{dx}\\ 0 \end{array}\right]\equiv N_{e}\;a_{e}-M_{q}. \end{array}$$

After applying Equations (43) and (45) and inserting the expressions for the shape functions, the compliance matrix of the 2M2f element can be expressed as follows:

(52) keTim,2M2f=keEB,2M2f+keQ,2M2f≡=13leEJ−145le3EJ1le16leEJ−7360le3EJ−1le−145le3EJ2945le5EJ+13lek13le−7360le3EJ3115120le5EJ+16lek16le1le13le0−1le16le016leEJ−7360le3EJ−1le13leEJ−145le3EJ1le−7360le3EJ3115120le5EJ+16lek16le−145le3EJ2945le5EJ+13lek13le−1le16le01le13le0+=1Ksle00−1Ksle000145le3Ks007360le3Ks0000000−1Ksle001Ksle0007360le3Ks00145le3Ks0000000

while the element column-vector of the right-hand-side terms takes the form

(53) $$\begin{array}{cc} f_{e}^{\mathrm{Tim},2M2f} & =f_{e}^{\mathrm{EB},2M2f}+f_{e}^{Q,2M2f}\equiv \left[\begin{array}{c} -\frac{1}{24}\frac{ql_{e}^{3}}{EJ}\\ \frac{1}{240}\frac{ql_{e}^{5}}{EJ}\\ \frac{1}{2}\;q\;l_{e}\\ -\frac{1}{24}\frac{ql_{e}^{3}}{EJ}\\ \frac{1}{240}\frac{ql_{e}^{5}}{EJ}\\ \frac{1}{2}\;q\;l_{e} \end{array}\right]+\left[\begin{array}{c} 0\\ \frac{1}{24}\;\frac{ql_{e}^{3}}{K_{s}}\\ 0\\ 0\\ \frac{1}{24}\;\frac{ql_{e}^{3}}{K_{s}}\\ 0 \end{array}\right]. \end{array}$$

In Equation (52), the non-zero terms occurring in the third and sixth rows and the third and sixth columns of the element matrix are inserted to satisfy the equilibrium of shear forces at nodes connecting elements. This remark also refers to the third and sixth rows of the element vector in Equation (53).

After implementation of the 2M2f element in a computer program, the problem related to the Euler–Bernoulli beam model can be solved by assuming that the value of the shear stiffness is very large. When the elastic foundation does not appear in the analysis, the second degree of freedom, $q_{r}$, should be set to zero for each node. The same notice is true in the case of element 2M1f.

## 5. Error Estimation of the Approximate Solution

The Synge hypercircle method [26] allows to estimate easily the error of the approximate solution when two dual solutions of a particular problem are available (the proposed solution minimizing the complementary energy functional and the displacement-based solution minimizing the potential energy functional). In the case of the Euler–Bernoulli beam, the functional of potential energy has the form

(54) $$\begin{array}{c} \Pi \left(w\right)=\frac{1}{2}\;\int_{0}^{l}\left[EJ{\left(\frac{d^{2}w}{dx^{2}}\right)}^{2}+k\;w^{2}\right]dx-\int_{0}^{l}q\;w\;dx-\bar{Q}\;w{|}_{\Gamma_{Q}}+\bar{M}\;\frac{dw}{dx}{|}_{\Gamma_{M}} \end{array}$$

and is minimized on the following set of kinematically admissible set of deflections:

(55) $$\begin{array}{c} V=\left\{w\in H^{2}\left(0,l\right):w=\bar{w}\;\mathrm{on}\;\Gamma_{w}\;\mathrm{and}\;\frac{dw}{dx}=\bar{\varphi }\;\mathrm{on}\;\Gamma_{\varphi }\right\}. \end{array}$$

For the Timoshenko beam, the functional of potential energy is expressed as

(56) $$\begin{array}{c} \Pi \left(w,\varphi \right)=\frac{1}{2}\;\int_{0}^{l}\left[EJ{\left(\frac{d^{2}w}{dx^{2}}\right)}^{2}+K_{s}{\left(\frac{dw}{dx}-\varphi \right)}^{2}+k\;w^{2}\right]dx-\int_{0}^{l}q\;w\;dx-\bar{Q}\;w{|}_{\Gamma_{Q}}+\bar{M}\;\varphi {|}_{\Gamma_{M}} \end{array}$$

and is minimized on the following set of kinematically admissible set of deflections and rotations:

(57) $$\begin{array}{c} V=\left\{\left[\begin{array}{c} w\\ \varphi \end{array}\right]\in {\left[H^{1}\left(0,l\right)\right]}^{2}:w=\bar{w}\;\mathrm{on}\;\Gamma_{w}\;\mathrm{and}\;\varphi =\bar{\varphi }\;\mathrm{on}\;\Gamma_{\varphi }\right\}. \end{array}$$

Symbol $H^{s}\left(0,l\right)$, s=1,2, used in Equations (55) and (57) denotes the Sobolev space of functions that are square integrable with their derivatives of order up to *s*.

Let *H* denote a Hilbert vector function space equipped with the inner product

(58) $$\begin{array}{c} {\left(\tau ,\sigma \right)}_{H}=\int_{0}^{l}\;\tau^{T}\;C\;\sigma \;dx \end{array}$$

and the norm

(59) $$\begin{array}{c} \left||\;\tau \;|\right|={\left[{\left(\tau ,\tau \right)}_{H}\right]}^{\frac{1}{2}} \end{array}$$

where the generalized stress vectors, τ and σ, are defined in Section 2.2 and Section 3.2 for the Euler–Bernoulli and Timoshenko beam models, respectively. The compliance matrix, C, is defined in Equation (17) or (43) depending on the beam model. It can be proved that [26]

(60) $$\begin{array}{c} ||\;\sigma^{\mathrm{ex}}-\sigma^{k}\;||\leq ||\;\sigma^{s}-\sigma^{k}\;||,\;||\;\sigma^{\mathrm{ex}}-\sigma^{s}\;||\leq ||\;\sigma^{s}-\sigma^{k}\;\left|\right| \end{array}$$

where $\sigma^{\mathrm{ex}}$, $\sigma^{s}$, and $\sigma^{k}$ denote the exact, statically admissible, and kinematically admissible solutions, respectively. The norm of the difference between the dual solutions appearing on the right-hand-sides of inequalities (60)—and being a measure of the approximate solution error—can be expressed as follows:

(61) $$\begin{array}{c} ||\;\sigma^{s}-\sigma^{k}\;\left|\right|=\sqrt{2\left(\Sigma \left(\sigma^{s}\right)+\Pi \left(\sigma^{k}\right)\right)} \end{array}$$

where $\Sigma (\sigma^{s})$ and $\Pi (\sigma^{k})$ are the calculated values of functionals of complementary and potential energy, respectively. The kinematically admissible stress vector, $\sigma^{k}$, is obtained through mapping A,

$$\begin{array}{c} \sigma^{k}=A\;u^{k} \end{array}$$

being the superposition of the kinematic relations and constitutive equations. In the case of the Euler–Bernoulli beam, this mapping and the displacement vector, u, can be written in the forms

(62) $$\begin{array}{c} A=\left[\begin{array}{cc} EJ & 0\\ 0 & k \end{array}\right]\;\left[\begin{array}{c} -\frac{d^{2}}{dx^{2}}\\ 1 \end{array}\right]=\left[\begin{array}{c} -EJ\;\frac{d^{2}}{dx^{2}}\\ k \end{array}\right],\;u=\left[\begin{array}{c} w \end{array}\right]. \end{array}$$

For the Timoshenko beam, mapping A and generalized displacement vector u look as follows:

(63) $$\begin{array}{c} A=\left[\begin{array}{ccc} EJ & 0 & 0\\ 0 & K_{s} & 0\\ 0 & 0 & k \end{array}\right]\;\left[\begin{array}{cc} 0 & -\frac{d}{dx}\\ \frac{d}{dx} & -1\\ 1 & 0 \end{array}\right]=\left[\begin{array}{cc} 0 & -EJ\;\frac{d}{dx}\\ K_{s}\;\frac{d}{dx} & -K_{s}\\ k & 0 \end{array}\right],\;u=\left[\begin{array}{c} w\\ \varphi \end{array}\right]. \end{array}$$

The values of the relative error of approximate solutions (kinematically and statically admissible ones) can be estimated as follows:

(64) $$\begin{array}{c} \Delta_{k}\equiv \frac{||\;\sigma^{\mathrm{ex}}-\sigma^{k}\;\left|\right|}{||\;\sigma^{\mathrm{ex}}\;\left|\right|}\leq \frac{||\;\sigma^{\mathrm{ex}}-\sigma^{k}\;\left|\right|}{\min\left(||\;\sigma^{s}\;||,||\;\sigma^{k}\;\left|\right|\right)}\leq \frac{||\;\sigma^{s}-\sigma^{k}\;\left|\right|}{\min\left(||\;\sigma^{s}\;||,||\;\sigma^{k}\;\left|\right|\right)}, \end{array}$$

(65) $$\begin{array}{c} \Delta_{s}\equiv \frac{||\;\sigma^{\mathrm{ex}}-\sigma^{s}\;\left|\right|}{||\;\sigma^{\mathrm{ex}}\;\left|\right|}\leq \frac{||\;\sigma^{\mathrm{ex}}-\sigma^{s}\;\left|\right|}{\min\left(||\;\sigma^{s}\;||,||\;\sigma^{k}\;\left|\right|\right)}\leq \frac{||\;\sigma^{s}-\sigma^{k}\;\left|\right|}{\min\left(||\;\sigma^{s}\;||,||\;\sigma^{k}\;\left|\right|\right)}. \end{array}$$

If the best approximation of solution $\sigma^{\mathrm{best}}$ is introduced as

(66) $$\begin{array}{c} \sigma^{\mathrm{best}}\equiv \frac{1}{2}\left(\sigma^{s}+\sigma^{k}\right) \end{array}$$

then the following equality holds:

(67) $$\begin{array}{c} ||\;\sigma^{\mathrm{ex}}-\sigma^{\mathrm{best}}\left|\right|=\frac{1}{2}||\;\sigma^{s}-\sigma^{k}\left|\right| \end{array}$$

and the upper and lower bounds for its relative error $\Delta_{\mathrm{best}}$ can be estimated as

(68) $$\begin{array}{c} \frac{1}{2}\;\frac{||\;\sigma^{s}-\sigma^{k}\;\left|\right|}{\max\left(||\;\sigma^{s}\;||,||\;\sigma^{k}\;\left|\right|\right)}\leq \Delta_{\mathrm{best}}\equiv \frac{||\;\sigma^{\mathrm{ex}}-\sigma^{\mathrm{best}}\;\left|\right|}{||\;\sigma^{\mathrm{ex}}\;\left|\right|}\leq \frac{1}{2}\;\frac{||\;\sigma^{s}-\sigma^{k}\;\left|\right|}{\min\left(||\;\sigma^{s}\;||,||\;\sigma^{k}\;\left|\right|\right)}. \end{array}$$

## 6. Numerical Examples

Several examples are presented in order to verify the accuracy of the proposed approach. For comparison, the calculations are made using the displacement-based techniques. In the case of the Euler–Bernoulli model, the well-known Hermite element with four degrees of freedom is utilized, where the deflection and its derivative are degrees of freedom at each node.

In the analyses for the Timoshenko beam model, the element with parabolic and linear interpolation functions for the deflection and cross-section rotation, respectively, is utilized. This five-degrees-of-freedom element is described, for instance, in [10,11] and will be identified in this section by symbol “3d2r.” The second displacement-based element used for the comparison is based on the linked interpolation approach [4], where two and three degrees of freedom are exploited for approximation of the deflection and rotation functions, respectively. Symbol “2d3r” will identify this element. The reduced form of this element, called “2d2r”, will also be utilized for which both the quantities, the deflection and rotation, are approximated using two degrees of freedom for each.

The convergence of the present approach is studied and compared with the displacement models mentioned above. The *a posteriori’* error estimation for the approximate solution is shown using the Synge method [26].

All the calculations are made with the help of the authors’ computer program written in FORTRAN 90 language. The gfortran compiler running under control of Linux operating system was utilized.

### 6.1. Two-Span Symmetric Beam Loaded Uniformly

**Figure 4:**
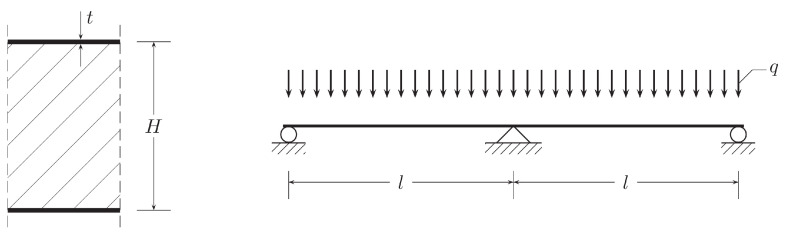
Bending problem for the Timoshenko beam.

As the first example, a three-layered steel-PUR-steel sandwich panel is considered. Its rectangular cross-section has height H=0.2 m and the unit width. The steel cladding thickness is t=0.75 mm. For steel, the Young modulus equals E=209 GPa. The poly-urethane core has the shear modulus G=4 MPa. A two-span symmetric beam with the span length l=6 m is considered as shown in Figure 4. The beam is loaded uniformly with distributed load q=0.5 kN/m. The above geometric and material data lead to the following characteristics of the panel cross-section: the inertia moment of the cross-section, $J=1.5\cdot 10^{-5}$ m${}^{4}$, and its shear rigidity, $K_{s}=800$ kN. Due to symmetry, only the right half of the beam is analyzed.

The stress-based elements 2M1f and 2M2f provide the same results. Three types of displacement-based elements mentioned above are employed in calculations for comparing the outcomes. The beam is analyzed with use of various numbers of elements: 5, 10, and 20 with equal lengths 1.2, 0.6, and 0.3 m, respectively.

**Figure 5:**
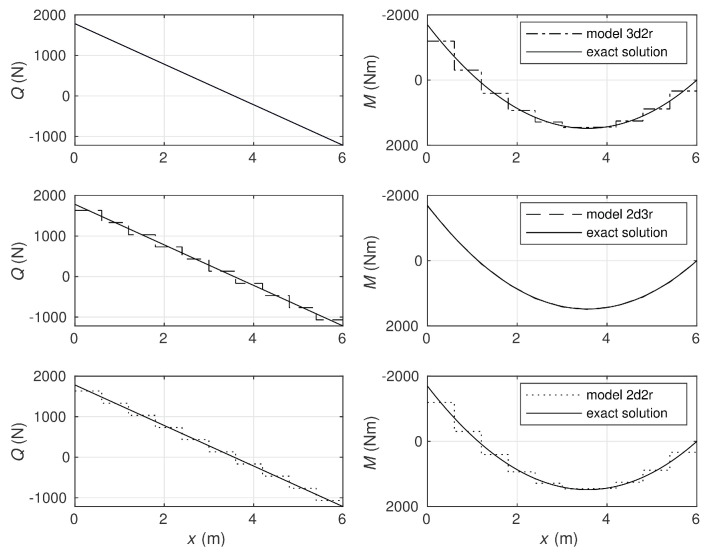
Comparison of section forces obtained by displacement-based models with the exact solution reproduced precisely by the equilibrium model.

Figure 5 presents a comparison of section forces of the beam obtained with use of all four Timoshenko beam models in the case of ten elements. In the following example, the equilibrium approach provides the exact solution regardless of the beam subdivision pattern. As the equilibrium method is able to reproduce the exact solution, it is seen that the shear locking phenomenon does not appear in the case of this approach. The displacement-based elements used for comparison of the calculation results also do not suffer from the shear-locking phenomenon. It follows from Figure 5 that the bending moment is approximated very precisely by the 2d3r model as well as the shear force by the 3d2r model, while the third displacement-based model, 2d2r, gives the piece-wise constant approximations for both the section forces.

**Figure 6:**
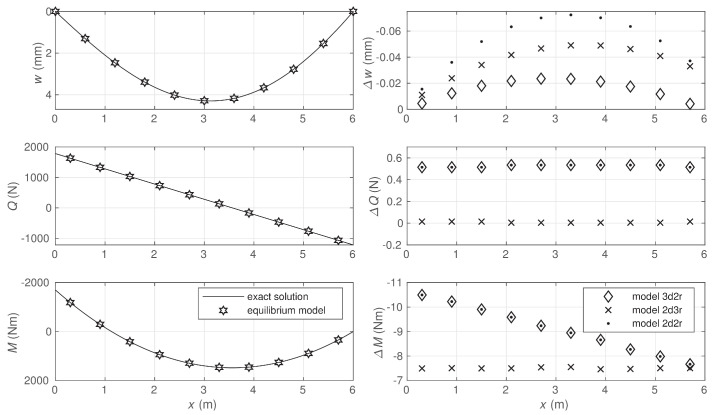
Comparison of deflections at nodes and section forces at central points of elements.

As the differences between the displacement-based solutions and the exact solution are very small for some quantities, these differences are shown directly for central points of elements in Figure 6. The 3d2r model approximates the deflection most precisely, while the 2d3r model gives the most precise approximates for the section forces. The equilibrium model has a possibility to determine deflections at nodes where the Lagrange multipliers are used to satisfy the equilibrium equation for the shear force. Exact values of the nodal deflections are obtained in the stress-based model.

**Figure 7:**
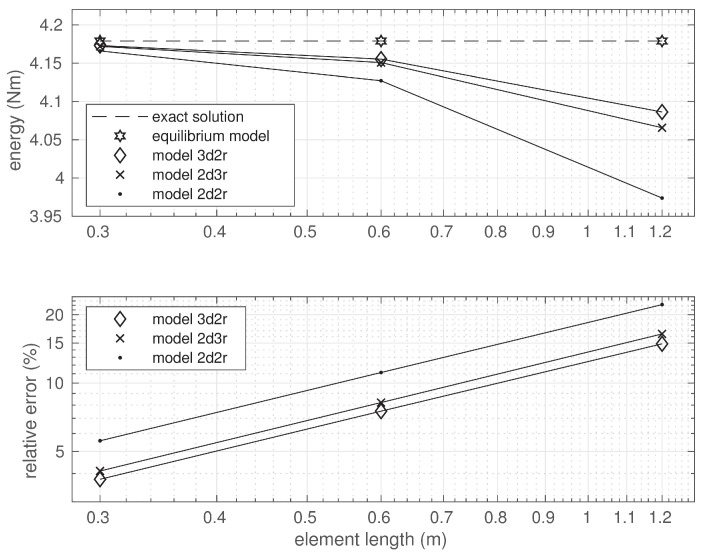
Strain energy and solution error.

All the displacement-based models used in the analysis satisfy the conditions of kinematic admissibility and provide the lower bounds for the value of the strain energy, which is shown in Figure 7.

In the case of models 3d2r and 2d3r, the strain energy values were significantly closer to the exact value than in the case of model 2d2r where both the functions approximating the deflections and rotations were linear. The results of the convergence study are shown in the same figure where the diagrams of relative energy errors are depicted. It is seen that all the utilized elements were characterized by the same order of convergence to the exact solution. However, the convergence rate differed due to different polynomial orders approximating the deflection and rotation functions. In the case of the finest mesh, the relative energy errors defined in Equation (64) were equal to 3.77%, 4.10%, and 5.57% for the solutions obtained by models 3d2r, 2d3r, and 2d2r, respectively.

### 6.2. Euler–Bernoulli Beam on Elastic Foundation

**Figure 8:**
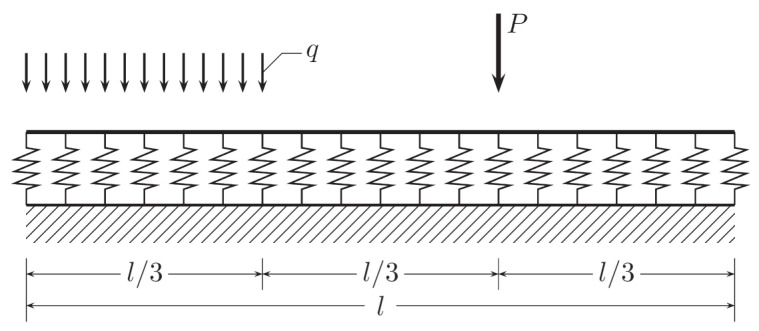
Beam on elastic foundation, problem definition.

The Euler–Bernoulli model of a beam resting on an elastic foundation is analyzed. The beam of length l=6 m is loaded by distributed and concentrated forces, $q=1\cdot 10^{4}\;N/m$ and $P=1\cdot 10^{5}\;N$, respectively, as shown in Figure 8.

It is assumed that the cross-section of the beam is a rectangle with the width and height equal to 0.2 m and 0.5 m, respectively. Calculations are made with the Young modulus $E=3\cdot 10^{10}$ Pa and the stiffness of the elastic foundation, 50$\cdot 10^{6}$ N/m${}^{3}$, which is equivalent to the Winkler constant $k=1\cdot 10^{7}$ N/m${}^{2}$. The lengths of beam elements applied in the calculations are l/3, l/6, l/12, l/24, and l/48.

**Figure 9:**
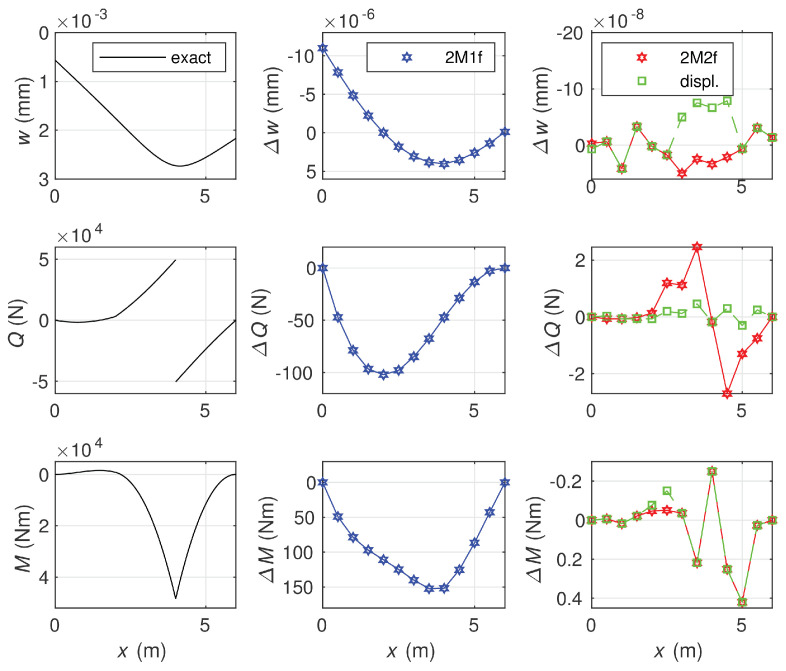
(**Left diagrams**): the deflection, shear force, and bending moment, the exact solution. The approximation solution errors for the deflection, shear force, and bending moment obtained by the 2M1f element (**middle diagrams**), and the 2M2f and the displacement-based element 2d3r (**right diagrams**).

The results of calculations obtained by use of the elements of length l/12 are shown in Figure 9.

As the differences between the equilibrium and displacement solutions are small, the diagrams of the deflection, shear force, and bending moment representing the exact solution are drawn on the left side of the figure, and the differences in these three quantities between the approximate solutions and the exact one are shown in the middle and on the right side of the figure. As the errors obtained for the 2M1f element were much larger than for other elements, they are displayed separately from the errors for the 2M2f and displacement-based elements. The reason for such differences is the order of the polynomial approximating the response of the elastic foundation. In the least accurate approximation case, it is a piece-wise constant function. Application of the piece-wise linear approximation of the foundation response in the equilibrium method increases the accuracy of the solution to the level comparable to the accuracy of the displacement-based method, where a piece-wise polynomial of the third degree is used. Calculation of nodal deflections in the equilibrium method is possible by use of the Lagrange multiplier method for enforcing equilibrium of the shear force at nodes.

**Figure 10:**
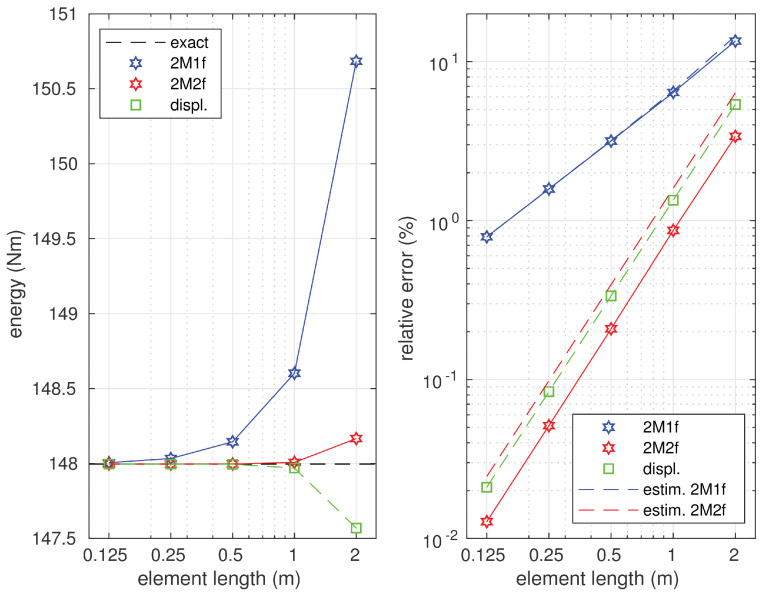
The lower and upper bounds for the strain energy (**left diagram**) and the errors of the approximate solutions (**right diagram**).

The equilibrium technique allows one to obtain the upper bound of the strain energy in contrast to its displacement counterpart. The values of the strain energy calculated by the three methods for several element lengths are represented on the left diagram in Figure 10.

They are compared with the exact value of the strain energy represented by the horizontal dashed line. The right diagrams in Figure 10 show the relations between the errors of the approximate solutions and the element length. These errors are represented by solid lines in the figure. The error estimations (by the Synge method) for the approximate solutions are also shown and depicted with the dashed lines. The estimated value of the error was slightly larger than the exact error of the equilibrium solution. The error analysis confirms the lower convergence order of the 2M1f element. It also shows that the convergence order was the same in the case of the two remaining elements; however, a more accurate approximation was observed when the equilibrium element 2M2f was applied.

### 6.3. Timoshenko Beam on Elastic Foundation

The same beam as in Section 6.2 is analyzed, but the Timoshenko model is considered. Computations are made with two values of the shear stiffness of the beam cross-section, $K_{s}$. In the first case, $K_{s}=1.0714\cdot 10^{9}$ N, which corresponds with the rectangular cross-section A=0.2·0.5=0.01 m${}^{2}$, the Poisson ratio $\nu =\frac{1}{6}$, and shape coefficient $\beta =\frac{6}{5}$,

$$K_{s}=\frac{E}{2\;(1+\nu )}\;\frac{A}{\beta }.$$

In the second variant of the analysis, an approximately five times smaller value is set for the shear stiffness, $K_{s}=0.2\cdot 10^{9}$ N. As in the previous Section, calculations are made for five cases of the element length l/3, …, l/48.

**Figure 11:**
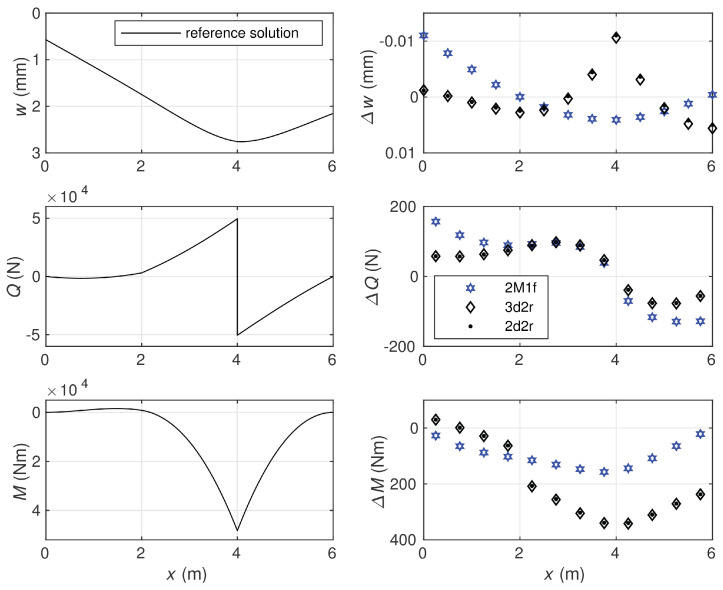
The reference solution: the deflection, shear force, and bending moment (**left diagrams**); comparison of these quantities obtained with 12 element meshes with the reference solution: for 2M1f, 3d2r, and 2d2r models (**right diagrams**). $K_{s}=1.0714\cdot 10^{9}$ N.

The results obtained for the first variant of data, $K_{s}=1.0714\cdot 10^{9}$ N, are presented in Figure 11, where the reference solution is shown in the form of plots for the beam deflection and the section forces.

The reference solution is determined by averaging the statically and kinematically admissible solutions obtained with a very fine element mesh containing 12,000 elements of equal length. The agreement for first eleven significant digits is obtained for the values of the strain energy calculated by exploiting the 2M2f and 2d3r elements. The differences between the deflections, shear forces, and bending moments calculated by use of various element types in the case of 12 element mesh and the corresponding reference quantities are shown in the figure. Smaller differences were observed for the bending moments obtained by the 2M1f equilibrium element than for those calculated by the two displacement-based ones: 3d2r and 2d2r. In the case of the shear forces, the relation was opposite. The comparable differences were obtained for the deflections when the three elements 2M1f, 3d2r, and 2d2r were exploited. It is seen that the displacement-based elements 3d2r and 2d2r produced very similar results.

**Figure 12:**
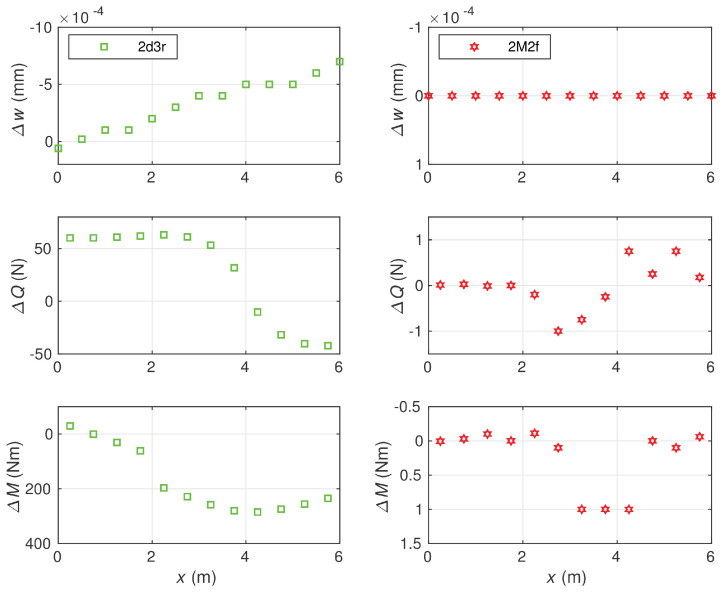
Comparison of errors for the deflections, shear forces and bending moments obtained with 12 element meshes: for 2d3r (**left diagrams**) and 2M2f (**right diagrams**). $K_{s}=1.0714\cdot 10^{9}$ N.

A comparison of differences related to more accurate elements, the 2M2f and 2d3r ones, is shown in Figure 12.

The node deflections obtained by the stress-based element 2M2f matched the reference values with respect to the first five significant digits, and their accuracy was significantly higher than the accuracy of displacement-based element 2d3r and other considered elements. In the case of section forces, a similar relation was observed. It follows from the diagrams that—comparing to the 2d3r element—the errors produced by the 2M2f element were about 300 times smaller for the bending moments and 50 times smaller for the shear forces.

**Figure 13:**
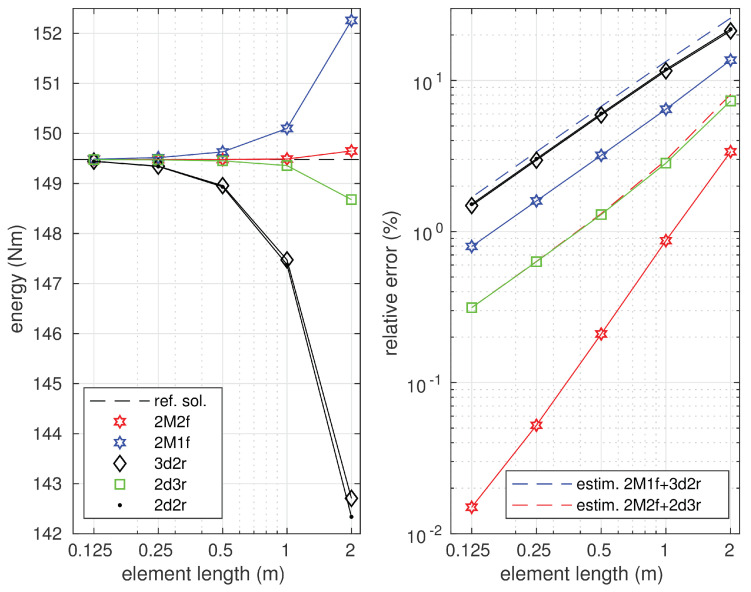
The strain energy and the errors of the approximate solutions in relations with the element length; $K_{s}=1.0714\cdot 10^{9}$ N.

The strain energy–element length relations are presented in Figure 13 for all the considered numerical models.

As expected, the equilibrium approaches provide the upper estimation for the strain energy value, while all the displacement-based elements estimate the strain energy value from below. All these results are compared with the reference value of the strain energy obtained as a mean of the values calculated using the equilibrium element 2M2f and linked interpolation element 2d3r with 12,000 equally sized elements. This reference energy value is indicated with the dashed line in the diagram.

The results of the error analysis presented in Figure 13 show that the stress–based 2M2f element had the fastest convergence. The linked interpolation element 2d3r had slower convergence than the 2M2f, while the two other displacement-based elements (3d2r and 2d2r) showed the slowest convergence. The equilibrium-based element 2M1f was placed on the third position. It is noticed that the convergence order of the elements 2Mf1, 2d3r, 3d2r and 2d2r was the same, while that of the 2M2f element was higher. The approximate solution errors estimated by the Synge method, using two combinations of the statically and kinematically admissible solutions, 2M1f + 3d2r and 2M2f + 2d3r, are indicated by the dashed lines plotted in blue and red color, respectively, in the same diagram. These estimations give slightly larger values than the actual errors of the displacement-based solutions.

**Figure 14:**
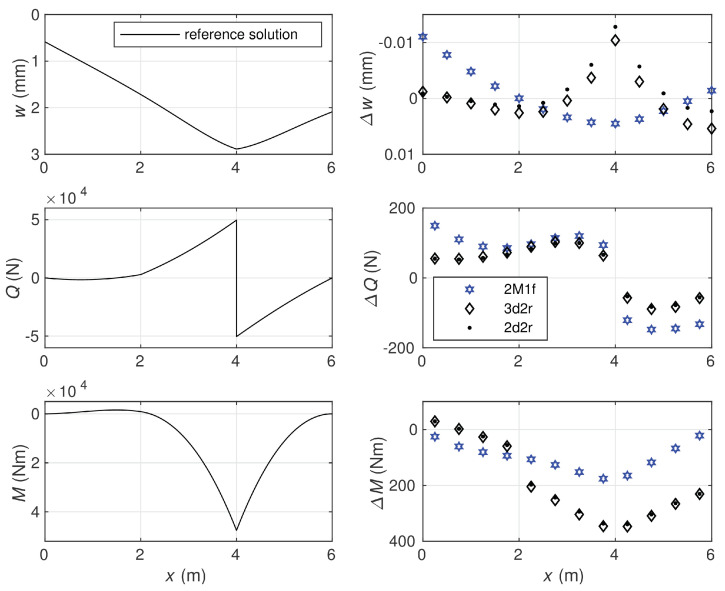
The reference solution: the deflection, shear force and bending moment (**left diagrams**); comparison of these quantities obtained with 12 element meshes with the reference solution: for 2M1f, 3d2r and 2d2r models (**right diagrams**). $K_{s}=0.2\cdot 10^{9}$ N.

**Figure 15:**
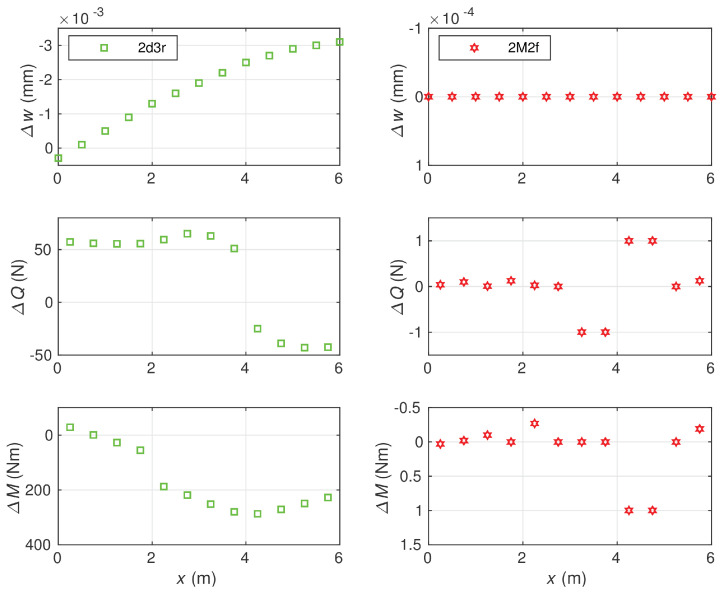
Comparison of errors for the deflections, shear forces and bending moments obtained with 12 element meshes: for 2d3r (**left diagrams**) and 2M2f (**right diagrams**). $K_{s}=0.2\cdot 10^{9}$ N.

The results of the analysis of the second variant of the beam with the smaller cross-section shear stiffness are shown in Figure 14 and Figure 15.

The reference solution presented in the left column of the diagrams in Figure 14 does not differ significantly from the results gained for the first variant of the calculations. The most visible difference is a sharper shape of the deflection diagram for the second case at the point where the concentrated load is applied. The larger error for the deflections are observed in the case of the 2d3r element in comparison to the previous data variant (Figure 15).

For other calculated quantities plotted in Figure 14 and Figure 15, similar magnitudes of the errors are are observed.

**Figure 16:**
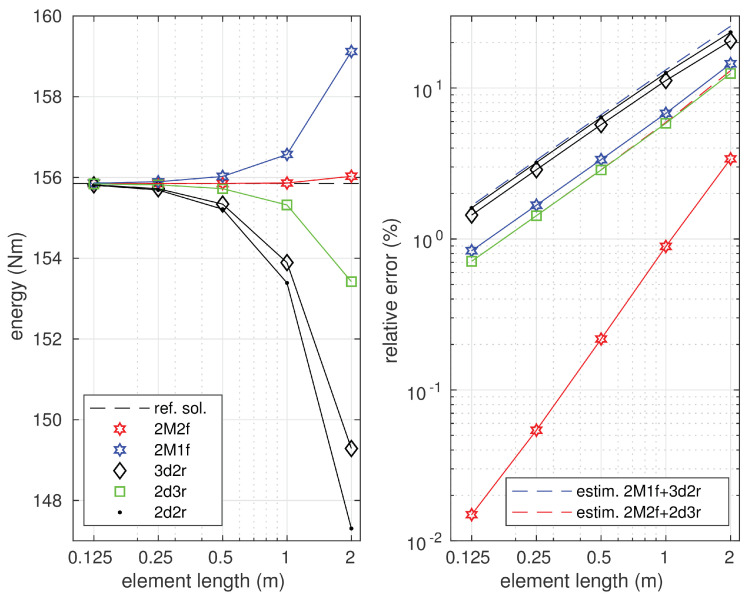
The strain energy and the errors of the approximate solutions in relations with the element length; $K_{s}=0.2\cdot 10^{9}$ N.

Again, the dependence of the value of the strain energy and the solution error on the element length is analyzed. The corresponding diagrams are presented in Figure 16.

Similar relations between the results related to various types of elements are observed as in the case of the first data variant. However, larger error values are noticed for the solution obtained by the linked interpolation element. An increase of the solution error is remarkable in the case of the 2d3r element and smaller in the case of the 2d2r element. The solution errors gained by other elements change only slightly comparing to the case of the larger cross-section shear stiffness.

## 7. Conclusions

Two elements for the Euler–Bernoulli and Timoshenko beams are successfully implemented in the stress-based format of the FEM. They can be used to solve static beam bending problems also in the case of interaction with the elastic foundation of Winkler type. Linear shape functions are used to approximate the function of bending moment in the elements. For the foundation response, the constant shape function is applied in the case of element 2M1f having five degrees of freedom, and the linear shape functions are employed in the case of the more accurate element 2M2f with six degrees of freedom. To conserve the equilibrium of the elements, the additional bending moments are added as the bubble functions, quadratic and cubic for the 2M1f and 2M2f elements, respectively. To fulfill the equilibrium for the shearing forces at nodes linking the neighboring elements, the Lagrange multiplier method is applied. This technique is applied by suitable modification of the element stiffness matrix and the element right-hand-side vector, which makes this technique very easy in implementation. The additional advantage of such an approach is a possibility to find deflections at nodes using the physical interpretation of the Lagrange multipliers.

Several numerical examples are presented. The results of of the stress-based FEM are compared with that obtained by three displacement-based finite element models. The error analysis is shown and on the base of the dual solutions, the statically and kinematically admissible ones, the error of approximate solution is estimated using the Synge method. Upper and lower bounds of the strain energy are obtained using the stress-based and displacement-based elements, respectively. Considering the two-span Timoshenko beam subjected to the uniformly distributed load, it is shown that the proposed stress-based element provides the exact solution of the problem, which means that the locking phenomenon does not appear in the stress-based approach.

In the case of the Euler–Bernoulli beam on an elastic foundation, a higher order of convergence is confirmed for the displacement-based elements comparing with the five-degree-of-freedom element 2M1f. The reason for that is the difference in the degree of polynomial approximating the foundation response, which is equal to three in the case of the displacement-based elements and zero in the case of the stress-based element. The six-degrees-of freedom element 2M2f has the same convergence order as the displacement-based elements, but it appears to be somewhat more accurate.

The calculations made for the Timoshenko beam resting on elastic foundation show that the equilibrium-based elements are more competitive than for the Euler–Bernoulli beam model. The 2M1f element reveals the same convergence order as the displacement-based elements and is more accurate than the 3d2r and 2d2r elements. The six-degrees-of-freedom element 2M2f shows a higher order of convergence than other elements.

The stress-based formulation of the FEM for the beam bending problem seems to be a little more complicated than its displacement-based counterpart. However, it has the advantageous possibilities and, therefore, it may be considered as an attractive tool in analysis of beam structures.

## Data Availability

Not applicable.
